# Immune checkpoint inhibitor‐related haemophagocytic lymphohistiocytosis in a patient with non‐small cell lung carcinoma

**DOI:** 10.1002/rcr2.1117

**Published:** 2023-03-08

**Authors:** Setsuko Oyama, Tatsuya Shirai, Yukiko Abe, Maya Tsuchiya, Toshiya Inui, Kozo Suhara, Satoshi Noto, Mitsuhiro Kamimura

**Affiliations:** ^1^ Department of Respiratory Medicine National Hospital Organization Disaster Medical Center Tokyo Japan; ^2^ Department of Rheumatology National Center of Global Health Tokyo Japan; ^3^ Department of Respiratory Medicine Kyorin University School of Medicine Tokyo Japan; ^4^ Department of Cardiology and Respirology Medicine Gifu University Graduate School of Medicine Gifu Japan; ^5^ Hematology Division National Hospital Organization Disaster Medical Center Tokyo Japan; ^6^ Department of Hematology Nerima Hikarigaoka Hospital Tokyo Japan

**Keywords:** haemophagocytic lymphohistiocytosis, immune checkpoint inhibitors, lung cancer

## Abstract

Hemophagocytic lymphohistiocytosis (HLH) has been reported as a rare complication of immune checkpoint inhibitors (ICI); however, ICI‐related HLH is a life‐threatening and comparatively late adverse event. Early diagnosis is critical, and it should be included in the differential diagnosis especially in patients with cytopenia with fever and hyperferritinaemia.

## Graphical abstract

Hemophagocytic lymphohistiocytosis (HLH) is a rare complication of immune checkpoint inhibitors (ICI), and moreover, presents several weeks or months after initiation of ICI treatment making it difficult to diagnose. We present a case, in whom pancytopenia with fever and hyperferritinaemia led us to suspect HLH. Early diagnosis of ICI‐related HLH is essential for a better outcome.

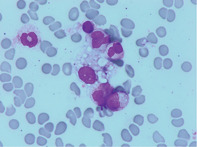

## CLINICAL IMAGE

**FIGURE 1:**
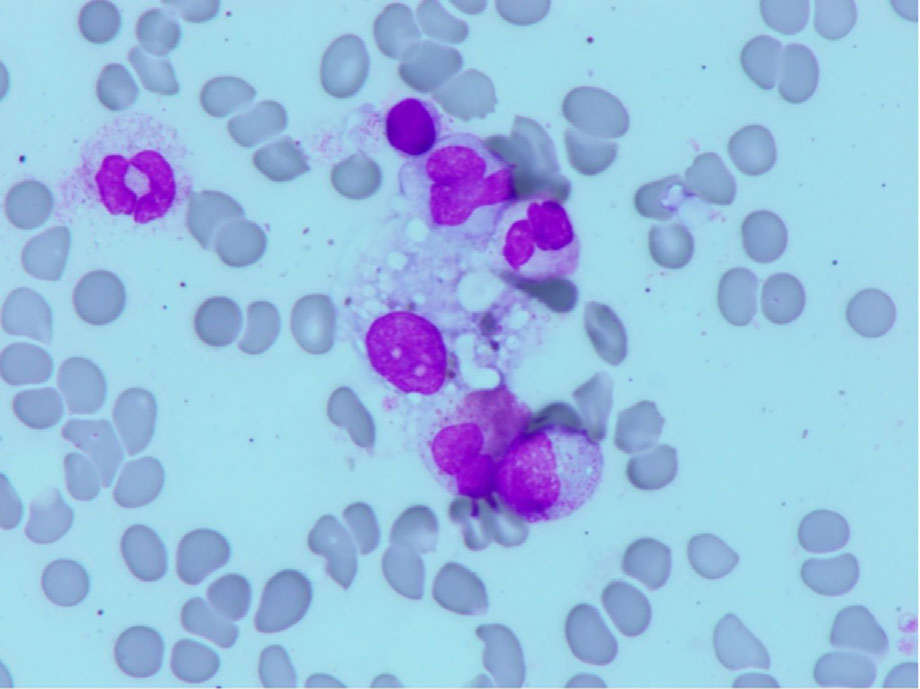
Bone marrow aspiration of our patient showing macrophages phagocytosing blood cells (May‐Grünwald–Giemsa staining)

A 60‐year‐old man with metastatic non‐small cell lung carcinoma was admitted to our hospital due to fever and loss of appetite. Seven months before admission, he received carboplatin, pemetrexed, and pembrolizumab, and subsequently received pemetrexed and pembrolizumab. He became febrile 6 days after the last pembrolizumab dose. Physical examination was unremarkable. Laboratory test results revealed pancytopenia and elevated ferritin level (9850 ng/dL) and soluble interleukin‐2 receptor level (3365 U/mL, reference range: 157–474). Bone marrow aspiration revealed haemophagocytosis (Figure 1). Extensive workups for autoimmune diseases and infectious diseases were negative. Diagnosis of pembrolizumab‐related haemophagocytic lymphohistiocytosis (HLH) was made based on HLH‐2004 guideline.^1^ Predonisolone at 1 mg/kg per day significantly improved his condition. Prednisolone was tapered with no recurrence of HLH during follow‐up for 11 months.

Recently, immune checkpoint inhibitor (ICI)‐related HLH was reported, and it can often be fatal.^2^ HLH is a rare complication of ICI, and moreover, presents several weeks or months after initiation of ICI treatment.^2^ Therefore, it is difficult to make a prompt diagnosis. In our case, pancytopenia with fever and hyperferritinaemia led us to suspect HLH. Additionally, performing bone marrow examination is very important for definite diagnosis. Early diagnosis of ICI‐related HLH is essential for a better outcome.

## AUTHOR CONTRIBUTION STATEMENT

Setsuko Oyama and Tatsuya Shirai drafted the manuscript. Satoshi Noto performed and analysed bone marrow aspiration. Tatsuya Shirai, Yukiko Abe, Maya Tsuchiya, Toshiya Inui, Kozo Suhara, and Mitsuhiro Kamimura cared for the patient. All the authors critically reviewed the manuscript and approved the final manuscript.

## CONFLICT OF INTEREST STATEMENT

None declared.

## ETHICS STATEMENT

The authors declare that appropriate written informed consent was obtained for the publication of this manuscript and accompanying images.

## Data Availability

Data sharing not applicable ‐ no new data generated
